# Toward a new conception of habit and self-control in adolescent maturation

**DOI:** 10.3389/fnhum.2014.00525

**Published:** 2014-07-25

**Authors:** Jose Víctor Orón Semper

**Affiliations:** Mind-Brain Group, Institute Culture and Society, Universidad de NavarraPamplona, Spain

**Keywords:** habit, self-control, adolescent, grit, emotion regulation

Neuropsychology shows us that adolescent maturation involves three areas: ejective functions, personal identity, and socialization and this maturation is not reached without emotion regulation. If we look at what this emotion regulation is made up of, psychology will tell us motivation, stress, resilience, emotional cognition, self-control, and habits are fields in which emotion regulation is useful. All of them are looking at the same thing but from different points of view. We can consider forming good habits as the outcome of reached emotional regulation by continued effort of self-control. Currently, neuroscience has seen habit like motor routine and for that reason links habits with corticostriatal pathways, but this is a narrow view of habit. In this opinion article we propose others cerebral process that fit better with a more general conception of the habit. This is developed during adolescence through emotion regulation, so education could be crucial to reach healthy or unhealthy habit.

## The frame of adolescence

Lately, certain singularities of adolescence have been presented. Lag between cortical and subcortical maturation could explain adolescence's behavior (Ernst et al., 2009; Somerville and Casey, 2010), but we also think this should be present with other transformations typical of the age they are related with self-control and habits.

Nowadays we can confidently say neuropsychological maturation of human beings, far from being closed in the early years of life, extends until the end of the second decade or more. The specific challenge of adolescence is split in three fields: executive functions, identity, and socialization (Crone and Dahl, 2012). Mental processes of executive functions are mainly supported by the prefrontal cortex (García et al., 2009; Delgado-Mejía and Etchepareborda, 2013). Identity and socialization interact with each other and mainly rest in default mode (Dennis and Thompson, 2013; Teicher et al., 2013). These systems work together (Smallwood et al., 2012; Chen et al., 2013), but it is not only the maturation of these systems but also, as we will see, a global maturation and change of the whole brain. Singularity of adolescence is that from that age, their maturation needs are not only a convenient environment and time, but also the youth need to make good decisions and have healthy life experiences. So at the end of adolescence, around middles twenties, we can find young adults or eternal adolescents (Blakemore, 2008; Choudhury et al., 2012; Crone and Dahl, 2012; Giedd, 2012) and emotion regulation is a key component for successful adolescence (Zins et al., 2005; Crone and Dahl, 2012). Knowing that being a teenager does not mean committing inevitably, risky actions. That is because it is not the same sensation seeking or risky actions. Belonging to a given age group neither forces us to commit risky actions, nor guarantee us to be sensible. Only self-control education guarantees us to be sensible (Romer et al., 2010). As we are going to see, all the cerebral systems which support personal maturation mature through adolescence. Nevertheless some systems, like default mode, continue to change throughout life (Campbell et al., 2013).

## Self-control and habits from psychology

Self-control makes reference to knowing how to deal with our impulses in relation to our long-term goals. On the one hand, this long-term orientation has to do with motivation aspects, and on the other hand, self-control is developed in a stressful or temptation environment. So, we can understand self-control is like the daily way to develop self-regulation (Duckworth et al., 2013b).

Habit can be understood more generally than neuroscience. Neuroscience usually understands habit as a repetition of a given behavior. This is a mechanistic vision. Habit makes reference to an internal state that we can reach through voluntary repetition, and favor to behave in a given way, if we want it to (Bernacer and Giménez-Amaya, 2013; Bernacer et al., 2014). This frees us to pay attention to all the processes and allows us to focus on other processes. So acquiring good habits allows adolescents to successfully transit to adulthood. During childhood and adolescence the named habit is “grit” (Duckworth, 2013; Tough, 2013), what reminds us the philosophical term of perseveration. Grit is a better predictor for success than quotient intelligence (Duckworth et al., 2010, 2013a). Another process that comes from psychology is self-concept. This makes us orientated to behave in one way (Dweck, 2000).

The reason to present self-control and habit together is because maintained self-control creates perseverance, or grit, which is a habit and favors self-control. Sometimes they are presented independently. For instance the experience of the sweet with children aged 4-years-old (Duckworth et al., 2013b) is seen like self-control, but it is evident that parents who bring up their children until 4-years-old, are the same who bring them up for the rest of their lives, where they create habits.

## A new proposal from self-control and habit in neuroscience

### About self-control

We have to consider several elements

1. Amygdala and accumbens activation. Amygdala by its relations with hippocampus and prefrontal cortex (Kobera et al., 2008) is part of the process of knowing how to wait and not to be hasty, and also for taking on disadvantages because there is a later reward (Pesoa, 2010). Accumbens by its relation with hypothalamus has resources which help to not fall into addiction (Hoebel et al., 2007). Moreover, accumbens by its relations with cortical and sub cortical regions is part of a process of knowing how to delay reward or give up a present good for a future greater good (Cardinal et al., 2002).
2. Traditionally, the reactive character of both nucleuses has been exaggerated, when indeed it is an “educate” reactivity. Glutamatergic projections from prefrontal cortex affect accumbens' dopaminergic receptors fixing one way to react when accumbens receive dopamine from ventral tegmental area and substantia nigra (Picciotto, 2013).
3. We need not forget orbitofrontal cortex, which makes a biological brake over received impulse subcortical. It allows the “fast way” more affective to integrate with the “slowly way” more rational—then the decision-making system works well (Cardinal et al., 2002; Roech et al., 2007; Sladky et al., 2013).
4. The decision making system uses frontoparietal net to make the decision and other operculocingular to keep the action (Fair et al., 2007).

### About habit

We can think of all changes in activation which free prefrontal cortex to be in charge of the given process and then work in other aspects of the same process or even others. These changes create tendencies to act.

5. Changing the component of each net and gaining specificity in a given activity (Fair et al., 2007; Dennis and Thompson, 2013).
6. One important area is medial prefrontal cortex, in where we store long-term assessments of our lived experiences. Moreover medial prefrontal cortex sends directly projections to premotor and motor areas. It is useful to not imitate who we are looking at and also to keep our initiative to decide when to act. So this area is highly related with our personalization (Isoda and Noritake, 2013). Hippocampus is more active for short-term, medial prefrontal cortex for long-term (Bonnici et al., 2012) and lateral and medial parietal for supporting our believes and self-concept because they are part of default mode. This system is active in the process of self-reference and consciousness (Mason et al., 2007; Fransson and Marrelec, 2008).
7. There is one event well-known as “switch backward” and it happens at the end of adolescence. This process frees prefrontal cortex from having to do everything. So it is free for working on other things. It reminds us the concept of habit of the present topic. We are going to number several of them:
  - Ventromedial of prefrontal cortex changes its activation to entorhinal and temporal cortex for leading attention and then affects to episodic codification (Schott et al., 2011);
  - Medial prefrontal cortex changes its activation to temporoparietal junction for mentalization and perspective taken (Crone and Dahl, 2012);
  - From anterior cingulate cortex to parietal and occipital for filtering what is irrelevant (Velanova et al., 2008);
  - From dorsomedial prefrontal cortex to superior and posterior temporal for distinguishing between physical cause and intentional cause (Pfeifer and Blakemore, 2012);
  - From medial prefrontal cortex to temporal cortex for self-concept (Sebastian et al., 2008);
  - From dorsolateral prefrontal cortex to anterior cingulate cortex for impulse control (Fair et al., 2007).

## Conclusion

We have hypothesized several cerebral changes than could support a widely idea of habit and self-control. And as the period when these processes are formed is during adolescence, we highlighted adolescence education. The issue is not whether they reach habits, they will get it, however the issue is what kind of habits they are.

In this opinion article, we have marked only some points to offer a broad view of habit and self-control. These assertions need to be contextualized therefore in a more general frame. It is also needed to make differences between emotion, cognition, decision making, and so on in order to integrate them into a singular action. So we need to think about how to relate functional levels to neuroanatomical ones. And we need to consider the differences of importance among neurotransmitters because their influence has multilevel explanation. All of this shows the complexity of habit and self-control

### Conflict of interest statement

The author declares that the research was conducted in the absence of any commercial or financial relationships that could be construed as a potential conflict of interest.
